# Improving functional annotation for industrial microbes: a case study with *Pichia pastoris*

**DOI:** 10.1016/j.tibtech.2014.05.003

**Published:** 2014-08

**Authors:** Duygu Dikicioglu, Valerie Wood, Kim M. Rutherford, Mark D. McDowall, Stephen G. Oliver

**Affiliations:** 1 Cambridge Systems Biology Centre & Department of Biochemistry, University of Cambridge, Sanger Building, 80 Tennis Court Road, Cambridge CB2 1GA, UK; 2 European Molecular Biology Laboratory European Bioinformatics Institute (EMBL-EBI) Wellcome Trust Genome Campus, Hinxton, Cambridge CB10 1SD, UK

**Keywords:** Pichia pastoris, Komagataella pastoris, industrial microbes, functional annotation, recombinant protein production

## Abstract

The research communities studying microbial model organisms, such as *Escherichia coli* or *Saccharomyces cerevisiae*, are well served by model organism databases that have extensive functional annotation. However, this is not true of many industrial microbes that are used widely in biotechnology. In this *Opinion* piece, we use *Pichia* (*Komagataella*) *pastoris* to illustrate the limitations of the available annotation. We consider the resources that can be implemented in the short term both to improve Gene Ontology (GO) annotation coverage based on annotation transfer, and to establish curation pipelines for the literature corpus of this organism.

## Highlights

• The current status of the *Pichia pastoris* genome is shown to lack extensive functional annotation.
• GO annotation transfer and literature curation pipelines improve the functional annotation of genomes.
• Pipelines and tools that can improve the annotation status of the genomes of *Pichia pastoris* and many industrial microbes are considered.
• Well-annotated genome sequences will facilitate the utilization of these microbes in a broader range of synthetic biology applications.

## A widely used, but relatively less studied, yeast

*Pichia* (*Komagataella*) *pastoris* is a favourite host organism for recombinant protein production in both industry and academia. The earliest studies, focusing on the crystal structure of the peroxisomes of *P. pastoris*, date back as far as 1975 [1]. The use of the alcohol oxidase promoter *AOX* was reported in 1985 [2], and Cregg *et al*. reported the development of *P. pastoris* as a host for DNA transformations in 1985 [3]. As a methylotrophic yeast, *P. pastoris* is able to use the reduced one-carbon compound methanol as its sole carbon and energy source via its methanol catabolism and assimilation pathways. Genes involved in these pathways, such as *AOX1*, *FLD1* and *FMD1* are sources of strong inducible promoters to enhance the expression of heterologous proteins for biotechnological applications. The popularity of this yeast as a tool is also promoted by its relative ease of genetic manipulation and cultivation, the presence of intracellular machinery to effect post-translational modifications of the expressed proteins including glycosylation and disulfide bond formation, its ability to efficiently secrete recombinant proteins when grown at high cell densities, and its strong preference for respiratory growth [4]. Furthermore, this yeast is also a favourite model organism in the study of organelle biology and autophagy [5]. However, as with many industrial species, the use of *P. pastoris* as a model organism, and its future development as a vehicle in synthetic biology, is impeded by shortcomings in the functional annotation of its genome.

The popularity of *P. pastoris* in industrial applications has yet to stimulate the production of a wealth of research data, and there are important gaps in our understanding of its molecular cell biology and physiology. For example, a recent search of PubMed (May 2014) identified only ∼4400 publications referring to *P.* (*K.*) *pastoris* compared to nearly 105 000 for *S. cerevisiae*. Although a new genus, *Komagataella*, was proposed for *P. pastoris* in 1994 [6], the research community has been slow to take up this name – as of May 2014, PubMed included only 15 publications using this genus name. This small body of literature illustrates the need for a community effort to improve the informational platform available for *P. pastoris* in order to support and inform the rational design of strains optimised for the production of heterologous proteins or other biotechnological products.

Biotechnological *P. pastoris* strains were reclassified using genome sequence analysis [7] at the same time as the initial genome sequence and annotation of two strains of *P. pastoris* (GS115 and DSMZ70382) were reported [8,9], shortly followed by the high-quality genome sequence of another strain (CBS7435) [10]. The complete genome sequence has made high-throughput approaches feasible, but fewer than ten experiments have been reported in the public gene expression repositories (ArrayExpress [11] and Gene Expression Omnibus [12]), perhaps because commercial microarray chips are not yet available. For this reason, the pioneering studies of Mattanovich and co-workers on the *P. pastoris* transcriptome exploited custom-designed sets of oligonucleotides based on gene predictions generated by the Integrated Genomics Company [13]. A recent study has exploited RNA sequencing technology to gain a deeper understanding of the physiological responses associated with different degrees of misfolding of human lysozyme in *P. pastoris* [14]. Several studies have investigated the proteomic and lipidomic response of *P. pastoris* to transgene overexpression [15–18] as well as its response to environmental variations at the metabolomic [19–21] and fluxomic levels [22,23].

*P. pastoris* metabolism has been simulated *in silico* through the use of small [24] and genome-scale [25–27] metabolic models. Such models give insights into the physiology of the yeast and provide a roadmap for optimising recombinant protein production and secretion. More data are now being collected at the transcriptomic, proteomic, and metabolomic levels. This information, as well data on the functional interactions of genes and proteins, should increase the accuracy and scope of *in silico* models. Thus, in the near future, the ability of these models to predict correctly the metabolic impact of manipulating the endogenous genes of this yeast, or expressing heterologous coding sequences should be greatly enhanced.

## Evaluation of the existing functional annotation

Biological networks contribute significantly to our understanding of yeast physiology. Physical interactions, genetic interactions, and transcriptional regulatory network data provide insight into how different cellular components work as parts of the whole and contribute to the proper functioning of metabolism. Only 14 physical interactions are documented for *P. pastoris* in the main public interaction databases [28,29] and there are no reports of genetic or transcriptional regulatory interactions.

*P. pastoris* has neither an extensive body of published experimental information on its biology, nor a dedicated Model Organism Database (MOD). Thus, information on the functions of its genes depends primarily upon resources that provide functional inference from other species, such as UniProt [30], RefSeq [31], Ensembl Genomes [32], STRING [33], and KEGG [34]. Of these, only UniProt has capacity for detailed literature curation of individual species.

The most widely used system for functional annotation is the GO. GO is a bioinformatics resource that uses structured controlled vocabularies to describe the molecular functions, biological processes, and cellular components associated with individual gene products, as well as supporting data analysis and integration [35]. GO annotations can be broadly classified as manually assigned or automatically generated. Although automated annotations are generally regarded as accurate, they tend to be less specific than manually curated annotations [36]. *P. pastoris* has 5040 annotated protein-encoding genes and, of these, 3532 are assigned with 17 002 GO terms via the Gene Ontology Annotation (GOA) project [37]. All but 21 annotations are automatically inferred, mainly from protein family membership (11 233). Grouping annotations by category (often referred to as a ‘GO slim’) can be used to assess the breadth of available GO annotation for an organism of interest (Table 1).

## Steps to improve functional annotation via community curation

Improved annotation breadth and depth can be achieved either by transferring specific annotations supported by experimental data from orthologues in a well-studied species to their unstudied counterparts, or by the curation of the literature corpus for the species. *P. pastoris* is ideally placed taxonomically to make annotation propagation from *S. cerevisiae* highly informative. *P. pastoris* was included in version EG17 of the Ensembl Genomes Database [32]. The Ensembl software platform contains modules to support comparative analysis and can provide orthology predictions via the Compara pipeline.

Improving the breadth of functional annotation so that *P. pastoris* can be used effectively in systems-wide approaches is a preliminary hurdle. Ultimately, the community of researchers using *P. pastoris* as their experimental organism of interest will require the data to be presented in their own publications to assess the similarities and differences to other yeast, and to make unique contributions to the growing body of functional annotation. To establish pipelines for the curation of experimental data, we have implemented the Generic Model Organism Database (GMOD)-compliant functional curation tool Canto (http://curation.pombase.org/) for *P. pastoris* and made it available for community use [38]. Canto is a web-based tool for curation and literature management, developed primarily for the community curation of *Schizosaccharomyces pombe* literature for inclusion in PomBase. However, Canto can be easily adopted by research communities studying other species. Using Canto, researchers can select their publications, indicate the genes studied, and assign Gene Ontology terms, phenotypes, modifications, and genetic or physical interactions to these genes. Annotations collected will be shared with public databases (initially GO [35], GOA [37], UniProt [30], Ensembl Genomes [32], and BioGRID [39]). The BIOLEDGE consortium, which aims to develop improved bioinformatics tools for biotechnology applications (http://www.bioledge.eu/), will conduct initial trials using Canto, and we hope to establish a pilot project after consultation with the community.

Examining the literature corpus for *P. pastoris* available through PubMed, we see an increasing trend in the utilisation of *P. pastoris* in research, mainly in the domain of recombinant protein production. Other specific subsets related to vaccine target proteins and non-host proteins associated with diseases or other pathologies (allergens, and venoms or toxins) contributed 8% of the total. We also observe an increase in the production of curatable publications that would contribute to the accumulation of scientific knowledge on this yeast. To maintain this momentum, it is crucial that the information produced is made accessible in order that it may be used to analyse future data and generate new hypotheses.

## Concluding remarks and future perspectives

This assessment of the curation status of the *P. pastoris* genome demonstrates that, despite its widespread use as a host for the expression of heterologous proteins and the efforts of a small but dedicated research community, our knowledge of the physiological capabilities of this organism is limited. This creates a bottleneck for the rational design of this yeast to accommodate the current needs of the biotechnology and pharmaceutical industries. A similar state of affairs applies to several other industrial microbes. However, to indicate a way forward, we have implemented pipelines and made tools available that will enable the *P. pastoris* research community to utilise existing data from other species to inform their ongoing research and to self-organise to provide distributed curation capacity. This will benefit all researchers that use *P. pastoris* as either an experimental organism or as a ‘chassis’ for synthetic biology applications; it should also assist investigators to seek funding for the establishment of a dedicated MOD for *P. pastoris.* Whatever the outcome of such a quest, the availability of the Canto curation tool [38], the inclusion of *P. pastoris* in the Ensembl Genomes resource [37], and its adoption as a Reference Proteome in 2014_01 release of UniProtKB should raise the profile and utility of this organism as a model and provide a platform for the integration of knowledge from the existing distributed resources. In addition, the *P. pastoris* community will have access to tools to annotate systematically their own literature using universal curation protocols and drive forward the need for a dedicated data resource. We trust that communities of researchers working with other industrial microbes will find these tools of use in improving the annotation status of their organisms of choice.

## Figures and Tables

**Table 1:** Breadth and depth of GO annotation of the *Pichia pastoris* genome^a^

Biological process term name	GO ID	GOA
Autophagy	GO:0006914	26
Ascospore formation	GO:0030437	0
Carbohydrate metabolic process	GO:0005975	122
Cellular amino acid metabolic process	GO:0006520	184
Cell wall organisation or biogenesis	GO:0071554	7
Chromosome organisation	GO:0051276	48
Chromosome segregation	GO:0007059	9
Cofactor metabolic process	GO:0051186	74
Conjugation with cellular fusion	GO:0000747	1
Cytokinesis	GO:0000910	2
Cytoskeletal organisation	GO:0007010	31
DNA metabolic process	GO:0006259	132
Establishment or maintenance of cell polarity	GO:0007163	2
Generation of precursor metabolites and energy	GO:0006091	58
Iron sulfur cluster assembly	GO:0016226	7
Lipid metabolic process	GO:0006629	112
mRNA metabolic process	GO:0016071	28
Meiosis	GO:0007126	3
Mitochondrion organisation	GO:0007005	22
Nucleobase-containing metabolic process	GO:0055086	186
Nucleocytoplasmic transport	GO:0006913	9
Peroxisome organisation	GO:0007031	19
Protein catabolic process	GO:0030163	56
Protein complex assembly	GO:0006461	37
Protein folding	GO:0006457	40
Protein glycosylation	GO:0006486	22
Protein modification by protein conjugation	GO:0070647	14
Protein targeting	GO:0006605	37
Regulation of mitotic cell cycle	GO:0007346	4
Ribosome biogenesis	GO:0042254	43
Signalling	GO:0023052	116
tRNA metabolic process	GO:0006399	72
Transmembrane transport	GO:0055085	218
Translation	GO:0006412	227
Transcription, DNA-templated	GO:0006351	237
Vacuolar transport	GO:0007034	5
Vacuole organisation	GO:0007033	3
Vesicle-mediated transport	GO:0016192	78
Vitamin metabolic process	GO:0006766	19
No GO slim annotation		3177
Total number of genes		5040

a *P. pastoris* GO annotation was from GOA version 125. Slimming was performed using the Generic GO Term Mapper (http://go.princeton.edu GO obo 10/01/2014)
